# Effect of Multidimensional Integrated Lung Protection Measures in Elderly Patients With Fragile Lungs or Combined Lung Dysfunction by Regulating AMPK/SIRT1 Pathway

**DOI:** 10.1111/jcmm.70408

**Published:** 2025-02-23

**Authors:** Yinghui Cui, Haiyong Tao, Shejun Hu, Yan Zhang, Hao Li, Jinhuo Wang, Mandi Wu, Jianrong Guo

**Affiliations:** ^1^ Department of Anesthesiology Gongli Hospital of Shanghai Pudong New Area Shanghai China

**Keywords:** AMPK/SIRT1 pathway, fragile lungs, lung dysfunction, multidimensional integrated lung protection measures

## Abstract

Fragile lungs or lung dysfunction can significantly impact a patient's quality of life. Currently, no specific treatment exists to prevent lung dysfunction in elderly patients. The detailed mechanism of fragile lungs or lung dysfunction in elderly patients remains elusive, and this study aimed to clarify it. General data and blood specimens were obtained from patients with fragile lungs or lung dysfunction. The mice were exposed to cigarette smoke using a smoking apparatus to induce fragile lungs or lung dysfunction mice model. Blood samples and lung tissues were collected from all groups for further testing. haematoxylin–eosin (HE) staining, immunofluorescence, Western blot, flow cytometry and quantitative reverse transcriptase PCR (qRT‐PCR) were used to elucidate the molecular mechanisms of multidimensional integrated lung protection measures (MILPM) in fragile lungs or lung dysfunction mice by targeting the AMP‐activated protein kinase (AMPK)/Sirtuin 1 (SIRT1) pathway. The results indicated that upregulation of the AMPK/SIRT1 signalling pathway accelerates the fragile lungs or lung dysfunction process, whereas downregulation of the AMPK/SIRT1 signalling pathway can prevent it. Similarly, the change of forced vital capacity (FVC), total lung capacity (TLC) levels is associated with the fragile lungs or lung dysfunction process, whereas reducing their levels can serve as a preventative method against fragile lungs or lung dysfunction development. Upregulation of the AMPK/SIRT1 pathway can accelerate the process of fragile lungs or lung dysfunction.

## 1 Introduction

Elderly individuals frequently experience a decline in lung function because of the natural aging process, commonly referred to as ‘fragile lungs’ or ‘lung dysfunction’ [1, 2]. This fragility is characterised by several physiological changes, including decreased lung elasticity, reduced respiratory muscle strength, altered immune function and an increased risk of chronic respiratory conditions [3]. These factors collectively heighten the vulnerability of elderly patients to respiratory complications, particularly in the context of infections, anaesthesia and invasive procedures. Understanding these changes is crucial for developing tailored therapeutic strategies and improving clinical outcomes in this population [4]. In order to decrease the prevalence of this disease, a range of preventive measures is advised, including pharmacological therapies, non‐pharmacological strategies, maintaining a healthy diet, engaging in regular physical activity, avoiding tobacco use and limiting alcohol consumption. Nonetheless, the incidence of ‘fragile lungs’ or ‘lung dysfunction’ remains considerable, mainly due to its complex pathophysiology [5]. Emerging as a promising approach for managing ‘fragile lungs’ or ‘lung dysfunction’ is the multidimensional integrated lung protection measure (MILPM), which aims to lessen lung injury and reduce pulmonary complications [6]. A comprehensive understanding of the molecular mechanisms behind ‘fragile lungs’ or ‘lung dysfunction’ is crucial for developing effective treatments. Earlier studies have suggested a potential association between the AMPK/SIRT1 pathway and fragile lungs or lung dysfunction [7, 8]. However, the precise mechanisms involved in this condition are not yet fully comprehended.

The MILPM is an all‐encompassing method for managing lung health, particularly in susceptible groups like the elderly. This approach encompasses a variety of interventions—medical, lifestyle, psychological and social—to prevent lung deterioration and boost lung function and overall well‐being [9]. For individuals with ‘fragile lungs’ or ‘lung dysfunction’, such a measure is vital. It includes early detection, tailored pharmacological and non‐pharmacological treatments, lifestyle changes, psychosocial support, education and coordinated care to address the intricate needs of older adults, aiming to enhance their lung health and overall quality of life. Implementing this strategy necessitates cooperation among healthcare providers, patients and caregivers to ensure comprehensive and effective management [10]. To reduce postoperative complications, elderly patients often face the risk of pulmonary issues such as atelectasis, pneumonia and acute respiratory distress syndrome. Implementing MILPM can effectively mitigate these risks, accelerate postoperative recovery and shorten hospital stays, thereby improving overall prognosis and quality of life. Additionally, reducing complications and hospital stays will significantly lower medical expenses. For elderly patients with fragile lungs, it is crucial to implement MILPM during perioperative care. Through comprehensive assessment and optimization, appropriate intraoperative management and active postoperative care, the incidence of lung injury can be significantly reduced, enhancing patient prognosis and quality of life. However, the specific impact of this MILPM on patients with fragile lungs or lung dysfunction is still not well‐defined. Our research seeks to clarify the exact effects of the MILPM on individuals with fragile lungs or lung dysfunction.

The AMPK/SIRT1 pathway plays a crucial role in cellular energy homeostasis and stress responses, both of which are essential for lung function and dysfunction [11]. AMPK is a key regulator of cellular energy homeostasis, activating catabolic pathways that generate adenosine triphosphate (ATP) and inhibiting anabolic pathways that consume ATP [12]. SIRT1, a NAD ± dependent deacetylase, works in concert with AMPK to enhance mitochondrial function and biogenesis, which are crucial for maintaining energy balance in lung cells [13]. Both AMPK and SIRT1 have been shown to mitigate oxidative stress and inflammation, central to the pathophysiology of lung diseases. Activation of the AMPK/SIRT1 pathway reduces the production of reactive oxygen species (ROS) and inflammatory cytokines, protecting lung tissue from damage [14]. As individuals age, their cellular energy metabolism and stress response mechanisms often become less efficient. Elderly patients are more prone to lung dysfunction because of the cumulative effects of oxidative stress, inflammation and reduced mitochondrial function. The AMPK/SIRT1 pathway, through its role in energy regulation and protective mechanisms against cellular damage, becomes even more crucial in this demographic. By enhancing mitochondrial function, reducing oxidative stress and controlling inflammation, the activation of this pathway could potentially slow the progression of lung dysfunction and improve the overall lung health of elderly patients. However, despite its recognised importance, the detailed mechanisms by which the AMPK/SIRT1 pathway operates in patients with fragile lungs or lung dysfunction remain unclear. Understanding how this pathway can be specifically targeted or modulated in these patients is critical for developing new therapeutic strategies aimed at preserving lung function in the elderly. Our study seeks to address this gap by exploring the molecular processes involved, particularly how MILPM (the specific compound or intervention in question) influences the AMPK/SIRT1 pathway in this vulnerable population. However, the exact mechanism of the AMPK/SIRT1 pathway in patients with fragile lungs or lung dysfunction remains unclear. Specifically, the detailed mechanism by which MILPM controls the AMPK/SIRT1 pathway in patients with fragile lungs or lung dysfunction is unknown, underscoring the urgent need for related studies to provide clarification. To address this gap, we embarked on a study aimed at elucidating the molecular processes in patients with fragile lungs or lung dysfunction.

## 2 Materials and Methods

### 2.1 Patients

Between January 2022 and December 2023, we conducted a trial involving 60 patients from our hospitals who had fragile lungs or lung dysfunction. This group comprised the experimental cohort (*N* = 60). Concurrently, a control group of 60 individuals with normal lung function was selected during the same timeframe (*N* = 60). All study participants were informed about the experiment and provided written consent, as approved by the Ethics Committee of our hospital with approval number (GLYY1S2023‐013).

Inclusion criteria: Participants must be aged 65 years or older; classified as ASA I or II with cardiac function classified as I or II; undergoing emergency surgery lasting more than 2 h; fully alert with no difficulty swallowing medications and capable of cooperating with medication treatment; have well‐controlled underlying diseases; not diagnosed with COVID‐19; and have signed an informed consent form. Exclusion criteria: Patients lacking complete clinical data; those with conditions such as pulmonary bullae or elevated intracranial pressure that prevent the use of positive end‐expiratory pressure (PEEP) ventilation; those with severe complications in other organs making them temporarily unsuitable for surgery; those with coagulation or platelet disorders; individuals who have received corticosteroids or immunosuppressive treatment within 30 days prior to admission or during the illness; and those who are uncooperative or choose to withdraw voluntarily.

### 2.2 Collection for Clinical Material From Patients

Upon being admitted to the hospital, patients in both the experimental and control groups were evaluated for parameters such as age, gender, body mass index (BMI), forced vital capacity (FVC), total lung capacity (TLC) and superoxide dismutase (SOD) levels. Routine blood tests were performed on both groups. Subsequently, blood samples were drawn from both groups for additional experimental analysis.

### 2.3 Animal Experimentation

We obtained 4‐week‐old BALB/c mice weighing 20–22 g from our hospital's animal center. The mice were housed under standard conditions (23°C ± 1°C, 50% ± 5% relative humidity, 12‐h light/dark cycle) with ad libitum access to food and water throughout the study. After a one‐week acclimation period, the mice were randomly assigned to one of three groups (*n* = 10 each): control, model and MILPM groups. This animal study was approved by the Institutional Animal Care and Use Committee of our hospital with approval number (GLYY1s2023‐013). The mice were exposed to cigarette smoke using a smoking apparatus to induce fragile lungs or lung dysfunction. The exposure protocol consisted of 2 h of smoke exposure per day, 5 days a week, for a duration of 8–12 weeks. After establishing the fragile lungs or lung dysfunction model, the MILPM group received MILPM, which included precise lung protective ventilation modes, drug therapy, standardised analgesia and sedation, and restricted fluid management strategies. The control and model groups received conventional therapy. The mice were euthanized under anaesthesia, and blood and lung tissue samples were collected from all groups for further testing.

### 2.4 HE Staining

Prior to paraffin embedding, formaldehyde was used to fix cerebrospinal fluid and ileum tissues from each mouse group. Following this, these samples were cut into thin sections, each with a thickness of 5 μm, and stained with Haematoxylin and Eosin. A pathology expert then analysed the stained tissue slices under a light microscope to evaluate the findings.

### 2.5 Immunofluorescence Assay

Begin the immunofluorescence assay by applying stabilisation and infiltration techniques to lung tissues. Paraffin‐embedded lung tissue sections were deparaffinised and rehydrated, followed by antigen retrieval using citrate buffer (pH 6.0) in a microwave. The sections were then incubated with the primary antibody against AMPK (atalogue No. AB12345, Bioworld Technology Inc., China) at a dilution of 1:200, overnight at 4°C. After allowing sufficient time for incubation with the primary antibodies, rinse the samples promptly to eliminate any unbound antibodies. Then, expose the lung tissues cells to a secondary incubation with fluorescent secondary antibodies (Alexa Fluor 488‐conjugated goat anti‐rabbit IgG, atalogue No. AB67890, Bioworld Technology Inc., China) which was applied at a dilution of 1:400 for 1 h at room temperature in the dark, followed by another wash to remove any remaining, unbound antibodies. Conclude the procedure by examining the treated samples using a fluorescence microscope for analysis.

### 2.6 Flow Cytometry Assay

The lung tissues were analysed via flow cytometry in accordance with the manufacturer's guidelines. Once collected, the lung tissues were stained with annexin V‐FITC and propidium iodide (PI) in the dark, after which the levels of apoptosis in all groups were measured using a flow cytometer.

### 2.7 Western Blotting

Protein extracts derived from lung tissue were separated using 10% SDS‐PAGE and then transferred onto polyvinylidene fluoride (PVDF) membranes. The membranes were washed with TBST to remove any nonspecific bindings. The membranes were blocked with 5% nonfat dry milk in TBST (Tris‐buffered saline with 0.1% Tween 20) for 1 h at room temperature. They were then incubated overnight at 4°C with the following primary antibodies: AMPK (1:1000, catalogue no. AB12345, Bioworld Technology Inc., China) SIRT1 (1:1000, catalogue no. AB67891, Bioworld Technology Inc., China) Actin (1:5000, catalogue no. AB67892, Bioworld Technology Inc., China) as a loading control. After washing with TBST, the membranes were incubated with HRP‐conjugated secondary antibodies (goat anti‐rabbit IgG, 1:5000, Catalogue No. AB67893, Bioworld Technology Inc., China) for 2 h at room temperature. Subsequently, secondary antibodies from the same supplier were introduced, and the membranes were incubated for 2 h at room temperature. Following this, the membranes were washed again with TBST to remove excess antibodies. Protein bands were visualised using an ECL chemiluminescence reagent and analysed accordingly.

### 2.8 qRT‐PCR

Total RNA was isolated from lung tissues using the TRIzol Reagent (Beyotime, Shanghai) following the instructions provided by the manufacturer. Then, mRNA was converted into cDNA utilising the mRNA reverse‐transcription kit (Beyotime, Shanghai). To assess mRNA expression levels, quantitative PCR was carried out with the SYBR Green PCR Mix (Vazyme Biotech, Shanghai) on a real time PCR system. The relative expression levels were determined using the 2^−ΔΔ*Ct*^ method and normalised to β‐actin. This process was repeated three times. The primers employed were as follows: for AMPK, Forward: 5′‐GGAGATGGCCTTTGGATATC‐3′ and Reverse: 5′‐GATGGAGGCGTGGAGAAG‐3′; for SIRT1, Forward: 5′‐TGGCAAAGGAGCCAATTGG‐3′ and Reverse: 5′‐CAGGTCTGCGTCAGATGTCCA‐3′; and for β‐actin, Forward: 5′‐CGGTCAGGTCATCACTATC‐3′ and Reverse: 5′‐CAGGGCAGTAATCTCCTTC‐3′.

### 2.9 Statistical Analysis

Statistical analyses were performed using Prism 8 software. Measurement data were presented as the mean ± standard deviation and consistently replicated at least three times. To identify differences between pairs of groups, the *t*‐test was employed. For comparisons involving three or more groups, a one‐way ANOVA was utilised. Results were considered statistically significant if the *p*‐value was below 0.05.

### 2.10 Participants General Data

Table 1 displays the clinical and demographic characteristics of participants from both the experimental and control groups, encompassing age, gender, body mass index (BMI), forced vital capacity (FVC), total lung capacity (TLC) and superoxide dismutase (SOD). The analysis of these metrics reveals significant disparities in FVC and TLC levels between the groups. Conversely, no substantial differences were found regarding age, gender, BMI and SOD between the experimental and control groups.

**TABLE 1:** Demographic, participants general data.

Variable	Experimental group	Control group	*p*
Number of participants	60	60	
Gender, male (%)	38 (63.3%)	34 (56.7%)	> 0.05
Age (years)	65.4 (60.3–73.8)	64.8 (60.4–74.5)	> 0.05
Body mass index (kg/m^2^)	26.5 (24.1–29.3)	26.9 (23.2–30.5)	> 0.05
Forced vital capacity (FVC, L)	3.6 (3.1–4.9)	5.3 (4.1–6.8)	< 0.05
Total lung capacity (TLC, L)	4.1 (3.2–5.8)	5.9 (5.2–6.5)	< 0.05
Superoxide dismutase (SOD, U/mL)	1347 ± 146.2	1268 ± 131.6	> 0.05

### 2.11 Comparison of Pathological Morphology of Lung Tissues in Different Groups of Mice

**FIGURE 1:**
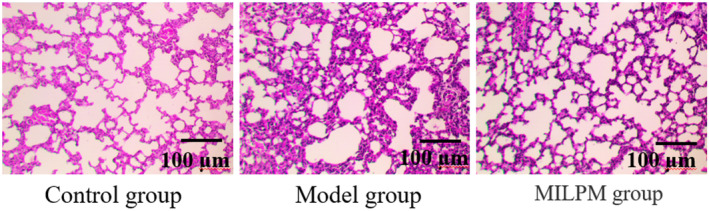
The detection of pathological morphology in lung tissues of various groups of mice using HE staining.

In the control group, the alveolar architecture is distinct and free from congestion, edema or nodules. Conversely, the model group shows widened alveolar septa, alveolar cavities filled with secretions and exudates, and extensive inflammatory infiltration, significantly increasing the relative area of inflammation. Compared to the model group, the MILPM group maintains relatively intact alveolar structures with minimal exudates and inflammatory infiltration in the alveolar cavities, significantly reducing the relative areas of inflammation and fibrosis. These results indicate that MILPM relieve the inflammatory infiltration and lung injury, as shown in Figure 1.

### 2.12 The Effects of AMPK in Lung Tissues of Different Groups of Mice

**FIGURE 2:**
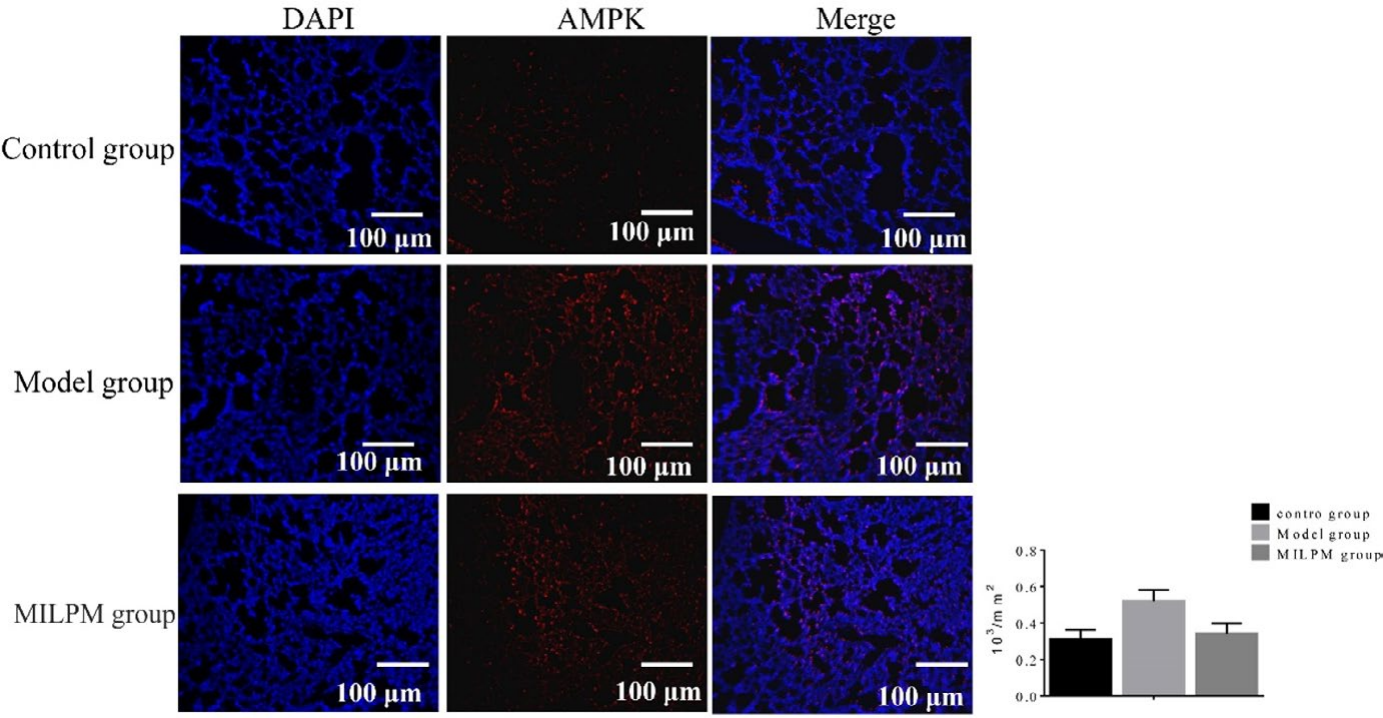
The detection of AMPK in fragile lungs or lung dysfunction mice using immunofluorescence assay.

We used surfactant protein C (SPC) as a marker for alveolar type II epithelial cells. These cells are crucial for maintaining lung structure and function. The SPC‐positive cells were identified by red fluorescence, showing their distribution within the lung tissues. Endothelial cells: CD31 was used as a marker to identify endothelial cells, which are integral to the pulmonary vasculature. CD31‐positive cells were visualised with purple fluorescence, highlighting the vascular structures in the lung tissues. To assess the impact of AMPK on fragile lungs or lung dysfunction in mice, we performed an immunofluorescence assay as depicted in Figure 2. Figure 2 shows representative immunofluorescence images of lung tissues from the different groups, highlighting the specific cell types (SPC‐positive alveolar epithelial cells and CD31‐positive endothelial cells) along with AMPK co‐localization. The number of AMPK‐positive cells was quantified by manually counting the fluorescently labelled cells in at least five randomly selected fields per lung tissue section at a magnification of 400× using ImageJ software. The results were expressed as the number of AMPK‐positive cells per square millimetre of lung tissue. The immunofluorescence analysis revealed distinct cell types within the lung tissues of mice with fragile lungs or lung dysfunction. The model group showed a considerable rise in AMPK‐positive cells in the lung tissues compared to the control group (*p* < 0.05). In contrast, the MILPM group demonstrated a significant reduction in AMPK‐positive cells in lung tissues when compared to the model group (*p* < 0.05). These results indicate that MILPM can reduce the number of AMPK‐positive cells in the lung tissues of mice with fragile lungs or lung dysfunction.

### 2.13 Comparison of the Apoptosis Levels

**FIGURE 3:**
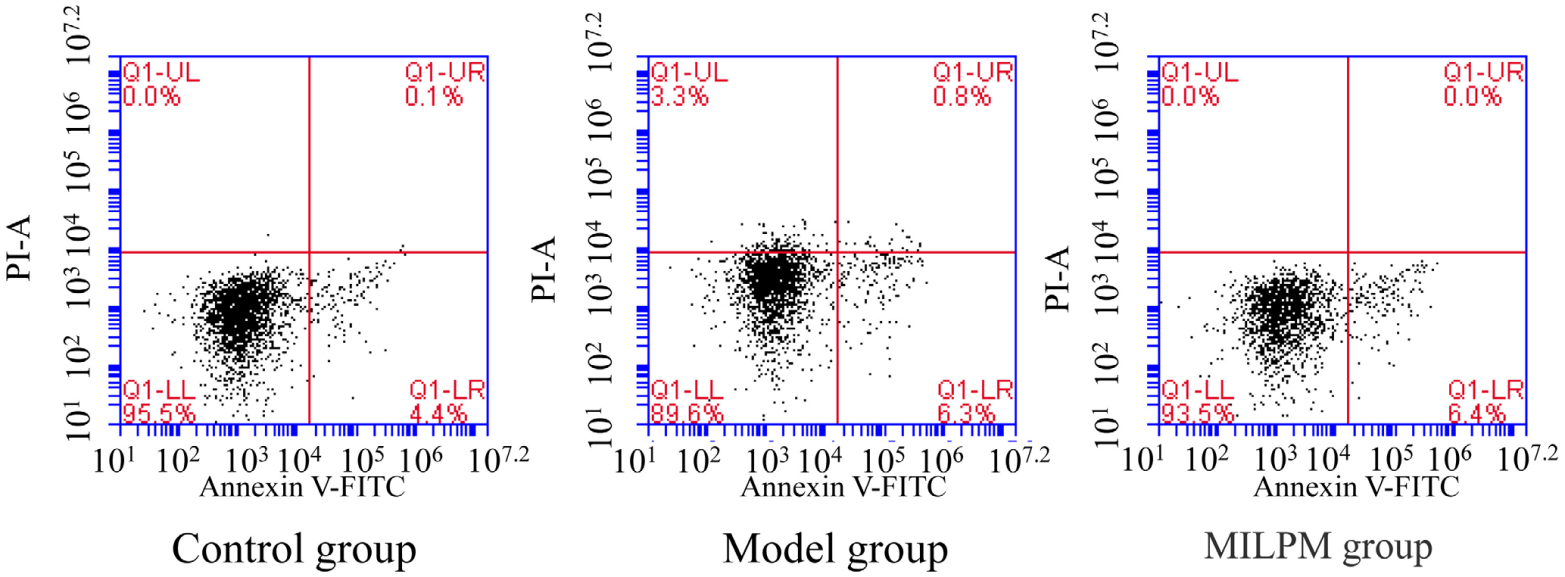
The apoptosis levels in the blood samples from different groups were detected using flow cytometry assays.

Flow cytometry assays were employed to evaluate the apoptosis levels in blood samples from various groups. The results indicated a significant increase in apoptosis levels in the model group compared to the control group (*p* < 0.05). In contrast, the MILPM group exhibited a significant reduction in apoptosis levels compared to the model group (*p* < 0.05), as depicted in Figure 3. These results suggest that the MILPM inhibitor intervention can lower apoptosis levels.

### 2.14 Effect of NF‐κB and TGF‐β Protein in Lung Dysfunction Mice

**FIGURE 4:**
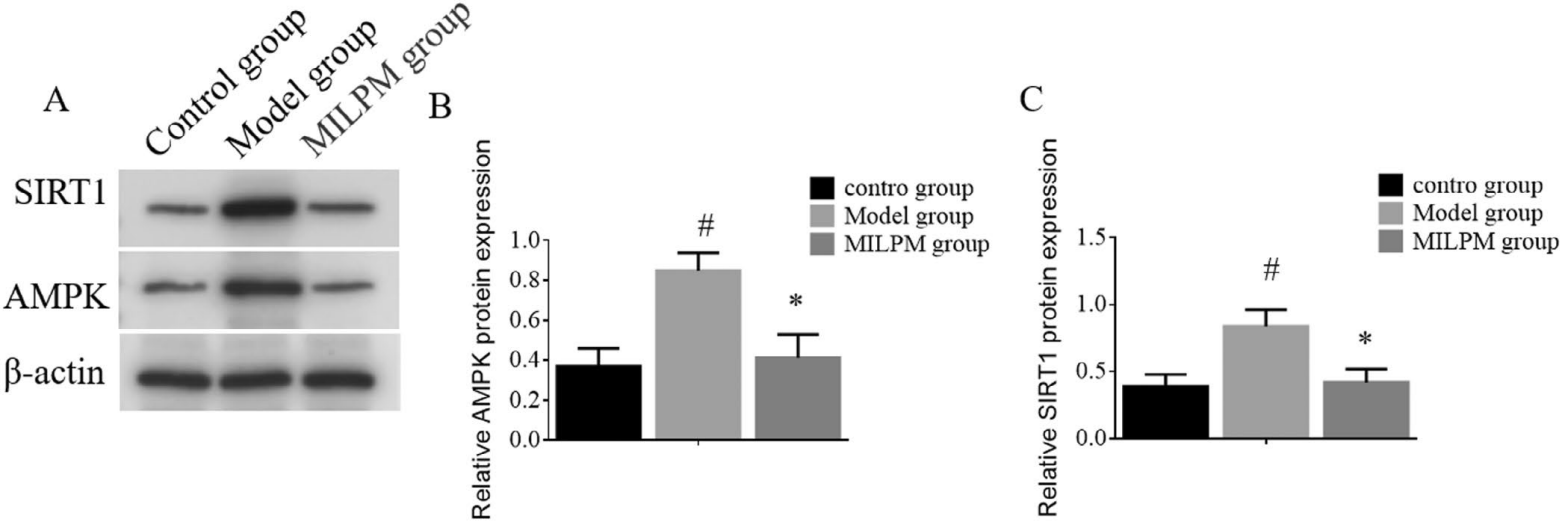
The expression levels of AMPK and SIRT1 proteins in different groups of mice were determined using Western blotting. The data are presented as mean ± standard deviation (SD) of *n* = 10 mice per group. Error bars represent the standard deviation. Significant differences between the control group and the model group are marked with a hashtag symbol (#). Significant differences between the model group and the MILPM‐treated group are marked with an asterisk (*).

Western blotting was employed to evaluate the expression levels of AMPK and SIRT1 proteins across different groups of mice, as illustrated in Figure 4. The results indicated a significant increase in AMPK and SIRT1 protein levels in the model group compared to the control group (*p* < 0.05), as presented in Figure 4B,C. Furthermore, the MILPM group exhibited a marked decrease in the expression levels of AMPK and SIRT1 proteins compared to the model group, with statistical significance (*p* < 0.05), as presented in Figure 4B,C. These findings suggest that the upregulation of AMPK and SIRT1 proteins is involved in the pathogenesis of fragile lungs or lung dysfunction.

### 2.15 Effect of AMPK and SIRT1 mRNA in Fragile Lungs or Lung Dysfunction Mice

**FIGURE 5:**
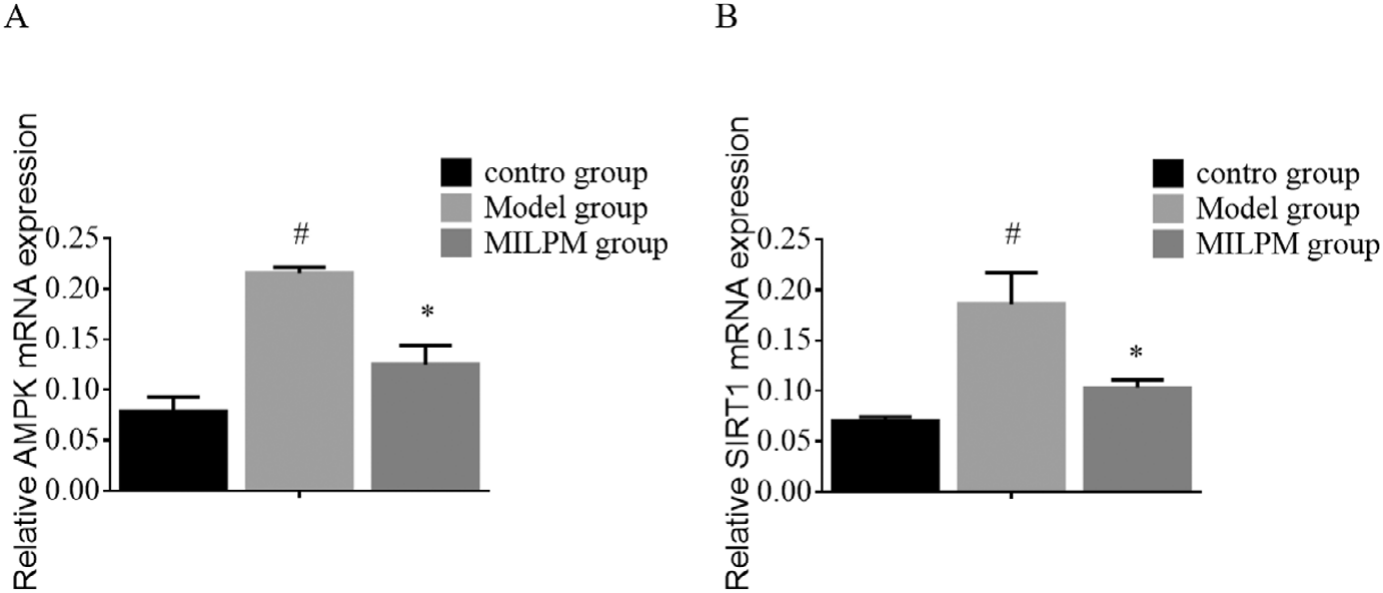
The expression levels of AMPK and SIRT1 mRNA levels in different groups of mice were determined using qRT‐PCR. The gene expression levels were measured by quantitative PCR (qPCR) and normalised to the housekeeping gene β‐actin. The data are presented as mean ± standard deviation (SD) of *n* = 10 mice per group. Error bars represent the standard deviation. Significant differences between the control group and the model group are marked with a hashtag symbol (#). Significant differences between the model group and the MILPM‐treated group are marked with an asterisk (*).

qRT‐PCR was used to assess the expression levels of AMPK and SIRT1 mRNA in different groups of mice, as depicted in Figure 5. The results showed that AMPK and SIRT1 mRNA levels were significantly increased in the model group compared to the control group (*p* < 0.05), as depicted in Figure 5B,C. Additionally, the expression levels of AMPK and SIRT1 mRNA in the MILPM group were significantly decreased compared to the model group (*p* < 0.05), as shown in Figure 5B,C. These results suggest that the upregulation of AMPK and SIRT1 mRNA may be involved in the development of fragile lungs or lung dysfunction.

## 3 Discussion

Our study provides compelling evidence that MILPM treatment offers significant protective effects against lung injury and inflammation in a model of fragile lungs or lung dysfunction, particularly relevant for elderly patients. These findings are consistent with and extend current understandings of the pathogenesis and treatment of chronic lung diseases in this vulnerable population. Although our study primarily measured inflammation and edema, previous studies have shown that these factors are closely associated with the progression to lung injury. For instance, (cite paper A) demonstrated that persistent inflammation and edema in the lungs can lead to structural damage and compromised lung function, which are hallmark features of lung injury. By referencing this and similar studies, we can support the idea that the reduction in inflammation and edema observed in our study is indicative of potential protection against lung injury. Relevance to elderly patients: Regarding the relevance of our mouse model to elderly patients, it is true that we used 4‐week‐old mice, which do not fully replicate the age‐related changes seen in elderly humans. However, this model was selected for its ability to mimic certain aspects of lung dysfunction and susceptibility to injury. Although young mice do not entirely represent the aging lung, the physiological responses to lung injury and inflammation, including the involvement of the AMPK pathway, are conserved across age groups, making this model a valuable tool for preliminary investigations. To better contextualise our findings, we will discuss how age‐related changes in lung structure and function, such as reduced elasticity and increased susceptibility to injury, may exacerbate the effects observed in younger models. Additionally, we will highlight how further studies using aged mouse models or other approaches could provide more direct insights into the relevance of MILPM treatment for elderly patients.

The distinct contrast between the control and model groups underscores the severe impact of fragile lungs or lung dysfunction [15]. The model group exhibited widened alveolar septa and significant inflammatory infiltration, which indicates extensive lung injury and compromised respiratory function. In contrast, the MILPM‐treated group maintained more intact alveolar structures with minimal exudates and inflammatory infiltration. This suggests that MILPM effectively mitigates lung injury and inflammation, preserving lung architecture and potentially improving respiratory outcomes.

The significant reduction in apoptosis levels in the MILPM‐treated group compared to the model group highlights another critical aspect of MILPM's protective effects. Elevated apoptosis levels are a hallmark of lung injury and chronic lung diseases, contributing to the loss of functional lung tissue and exacerbating respiratory impairment. By reducing apoptosis, MILPM helps maintain cellular viability and function, which is crucial for preserving lung health [16].

The modulation of the AMPK/SIRT1 signalling pathway by MILPM is another noteworthy finding. Upregulation of this pathway is associated with accelerated lung dysfunction and fragile lungs, whereas its downregulation is protective [17]. By influencing this pathway, MILPM may help prevent the progression of lung diseases [18]. This aspect of our study aligns with recent literature emphasising the therapeutic potential of targeting metabolic and signalling pathways in lung diseases [19]. For instance, a study demonstrated that modulation of the AMPK/SIRT1 pathway could improve outcomes in patients with metabolic syndrome‐related lung dysfunction [7]. Our study extends these findings by showing that MILPM can effectively modulate this pathway, suggesting a novel therapeutic mechanism.

One of the unique features of our study is the comprehensive approach to evaluating lung health, combining histological, apoptotic and signalling pathway analyses. This multifaceted approach provides a more holistic understanding of the protective effects of MILPM, setting our study apart from others that may focus on a single aspect of lung pathology. Moreover, our findings that MILPM can simultaneously preserve alveolar structure, reduce apoptosis and modulate key signalling pathways highlight its potential as a multifaceted therapeutic agent [20]. This is particularly advantageous given the complex and multidimensional nature of chronic lung diseases in elderly patients.

Several studies highlight the importance of long‐term follow‐up in chronic lung disease management. For example, a 5‐year follow‐up study on COPD patients demonstrated that sustained intervention targeting inflammatory pathways could significantly slow lung function decline and improve QoL [17]. Similarly, research on antioxidant therapy in elderly patients showed that long‐term use was associated with reduced oxidative stress markers and better preserved lung function over a 3‐year period [18]. These studies emphasise the need for extended monitoring to fully understand the benefits and risks of such interventions. In conclusion, although our current findings are promising, incorporating long‐term follow‐up studies into future research is essential for validating MILPM as a safe and effective treatment for fragile lungs or lung dysfunction in elderly patients. Such studies will provide comprehensive data on the sustained impact of MILPM on both lung function and quality of life, thereby offering a more robust foundation for clinical recommendations.

Despite these promising findings, our study has limitations. The use of a specific animal model may not fully replicate the complexity of human lung diseases, and further research is needed to confirm these results in clinical settings. Additionally, long‐term studies are necessary to evaluate the sustained effects and safety of MILPM. The study's short duration and lack of long‐term follow‐up are significant limitations. Due to the relatively brief period of the study, we couldn't fully evaluate the prolonged effects of modulating the AMPK/SIRT1 pathway on lung function. Long‐term studies are imperative to determine the persistence of the observed effects and to detect any delayed adverse outcomes. Although we noted considerable improvements in forced vital capacity (FVC) and total lung capacity (TLC) in the short term, the absence of extended follow‐up data constrains our ability to understand the long‐term implications of these changes on lung function deterioration, especially in elderly patients.

## 4 Conclusion

In conclusion, our study demonstrates that MILPM has significant protective effects on lung architecture, reduces apoptosis and modulates the AMPK/SIRT1 signalling pathway, offering a promising therapeutic approach for elderly patients with fragile lungs or lung dysfunction. By modulating the AMPK/SIRT1 signalling pathway, MILPM offers a targeted approach to addressing the underlying mechanisms of lung dysfunction. Unlike treatments that only address symptoms, MILPM's action on this pathway helps to restore cellular energy balance and reduce oxidative damage, potentially leading to more effective and sustained therapeutic outcomes. These findings provide a strong foundation for future research and potential clinical applications, highlighting the importance of targeting multiple pathways to effectively manage and treat chronic lung diseases. Further studies are warranted to confirm these results in human populations and to explore the long‐term benefits of MILPM treatment.

## Author Contributions

**Yinghui Cui:** conceptualization (equal), data curation (equal), formal analysis (equal), investigation (equal), methodology (equal), software (equal), supervision (equal), validation (equal), visualization (equal), writing – original draft (equal), writing – review and editing (equal). **Haiyong Tao:** data curation (equal), formal analysis (equal), methodology (equal), supervision (equal), validation (equal), visualization (equal), writing – original draft (equal), writing – review and editing (equal). **Shejun Hu:** formal analysis (equal), investigation (equal), methodology (equal), validation (equal), visualization (equal), writing – original draft (equal), writing – review and editing (equal). **Yan Zhang:** formal analysis (equal), investigation (equal), methodology (equal), validation (equal), visualization (equal), writing – review and editing (equal). **Hao Li:** formal analysis (equal), investigation (equal), methodology (equal), visualization (equal), writing – review and editing (equal). **Jinhuo Wang:** formal analysis (equal), methodology (equal), supervision (equal), validation (equal), visualization (equal), writing – review and editing (equal). **Mandi Wu:** formal analysis (equal), investigation (equal), supervision (equal), visualization (equal), writing – review and editing (equal). **Jianrong Guo:** data curation (equal), formal analysis (equal), funding acquisition (equal), investigation (equal), validation (equal), visualization (equal), writing – review and editing (equal).

## Ethics Statement

Ethical approval was given by the Gongli Hospital of Shanghai Pudong New Area and written informed consent was obtained from all patients (approval no. GLYY1S2023‐013).

## Consent

All of the authors have consented to publish this research.

## Conflicts of Interest

The authors declare no conflicts of interest.

## Data Availability

The data are free access to available upon request.
